# Pros and Cons of Pharmacological Manipulation of cGMP-PDEs in the Prevention and Treatment of Breast Cancer

**DOI:** 10.3390/ijms23010262

**Published:** 2021-12-27

**Authors:** Patrizia Di Iorio, Maurizio Ronci, Patricia Giuliani, Francesco Caciagli, Renata Ciccarelli, Vanni Caruso, Sarah Beggiato, Mariachiara Zuccarini

**Affiliations:** 1 Department of Medical, Oral and Biotechnological Sciences, University of Chieti-Pescara, 66100 Chieti, Italy; patrizia.diiorio@unich.it (P.D.I.); patricia.giuliani@unich.it (P.G.); francesco.caciagli@unich.it (F.C.); renata.ciccarelli@unich.it (R.C.); sarah.beggiato@unich.it (S.B.); 2 Center for Advanced Studies and Technologies (CAST), University of Chieti-Pescara, 66100 Chieti, Italy; maurizio.ronci@unich.it; 3 Department of Pharmacy, University of Chieti-Pescara, 66100 Chieti, Italy; 4 School of Pharmacy and Pharmacology, University of Tasmania, Hobart, TAS 7005, Australia; vanni.caruso@utas.edu.au

**Keywords:** cyclic GMP (cGMP), PKG, nitric oxide (NO), soluble guanylate cyclase (sGC), breast cancer, phosphodiesterase (PDE), chemoprevention, cyclooxygenase 2 (COX-2)–inhibitors, targeted therapy, drug repurposing

## Abstract

The cyclic nucleotides, cAMP and cGMP, are ubiquitous second messengers responsible for translating extracellular signals to intracellular biological responses in both normal and tumor cells. When these signals are aberrant or missing, cells may undergo neoplastic transformation or become resistant to chemotherapy. cGMP-hydrolyzing phosphodiesterases (PDEs) are attracting tremendous interest as drug targets for many diseases, including cancer, where they regulate cell growth, apoptosis and sensitization to radio- and chemotherapy. In breast cancer, PDE5 inhibition is associated with increased intracellular cGMP levels, which is responsible for the phosphorylation of PKG and other downstream molecules involved in cell proliferation or apoptosis. In this review, we provide an overview of the most relevant studies regarding the controversial role of PDE inhibitors as off-label adjuvants in cancer therapy.

## 1. Introduction

In accordance with the latest epidemiological data, breast cancer is the most frequently diagnosed cancer in women [1]. Despite early diagnosis due to advanced technologies and awareness-raising campaigns, breast cancer represents the second most common cause of cancer death in women worldwide [2]. The choice of pharmacological treatments depends on many factors, including histologic grade, estrogen/progesterone receptor (positive vs. negative) and human epidermal growth factor 2 (ERBB2) amplification, lymphovascular spread, organ sites of metastasis, comorbidities, menopausal status and age [3,4]. Importantly, genetic profiling allows a better stratification of tumors based on the different biological subtypes, leading to a more tailored therapeutic strategy.

The management of breast cancer comprises chemotherapy, hormone therapy, immunotherapy, and radiation, either as neo-adjuvant or adjuvant therapy (for a comprehensive review please refer to [5,6]. 

Cyclic guanosine monophosphate (cGMP) is a ubiquitous second messenger generated from GTP by two guanylate cyclases (GCs), namely a membrane-bound particulate GC (pGC) and a cytosolic soluble GC (sGC), in response to natriuretic peptides or nitric oxide (NO), respectively [7]. Within cells, cGMP activity is terminated by phosphodiesterases (PDEs). The latter is a class of hydrolases deputed to cAMP and cGMP breakdown into the inactive 5′-AMP or GMP. In human tissues, 21 genes encode at least 100 PDE splicing variants as a result of different gene expression and post-translational protein modifications [8]. PDEs are subdivided into 11 families differing each other for tissue distribution, inhibitory sensitivity and substrate selectivity (see Supplementary Table S1): PDE4/7/8 have affinity for cAMP, PDE5/6/9 are specific for cGMP, and PDE1/2/3/10/11 hydrolyze both cAMP and cGMP [9]. Therefore, cGMP concentration depends, among others, on: (i) cAMP- and cGMP- dependent activity of PDE1, PDE2, PDE10 and PDE11; (ii) cGMP-dependent activity of PDE2, PDE5, PDE6, and PDE9; and (iii) cAMP-dependent activity of PDE1 and PDE3 [10].

Cyclic GMP is sensed by receptor proteins containing cyclic nucleotide-binding (CNB) domains (i.e., the cGMP-dependent protein kinase G or PKG), and cyclic nucleotide gated (CNG) ion channels, by which it regulates ion flux [11]. PKG-I (α and β isoforms) and PKG-II are two kinases that regulate the localization and the function of many cellular proteins upon the phosphorylation of serine and threonine residues [12].

Depending on upstream or downstream effectors, cGMP mediates various biological effects, including vascular tone, neuronal functions, natriuresis, platelet activation, mitochondrial biogenesis, cell viability, cardioprotection and skeletal remodeling [13,14,15].

A significant number of studies reported that the dysregulation of cGMP/PKG signaling pathway accounts for numerous pathologies, including vascular and ventricular dysfunction, fibrosis, neurodegenerative disorders, hypertrophy and cancer [16,17,18,19].

A cyclic GMP/PKG signaling cascade can be triggered among other causes, by NO, a multi-faceted compound eliciting often opposite biological effects in different organ systems [18,20,21]. NO is an inorganic free radical gas synthesized via the oxidation of L-arginine by nitric oxide synthase (NOS), which exists in three isoforms, Ca^2+^/calmodulin-dependent neuronal NOS (nNOS or NOS1) and endothelial NOS (eNOS or NOS3), and Ca^2+^/calmodulin-independent inducible NOS (iNOS-NOS2) [22]. NO is also released from immune cells (neutrophils and macrophages) as a mechanism of defense against infections and tumor cells and its increase may support mutagenesis and cancer development [23].There are numerous molecular mechanisms through which NO may facilitate tumorigenesis, including direct DNA breaks through formation of reactive oxygen species (ROS) (NO reacts with superoxide and generates peroxynitrite) and genotoxic alkylating agents, as well as the inhibition of DNA-repairing enzymes, such as alkyltransferase and DNA ligase [24]

Both NO and cGMP participate in the signaling pathways involved in tumor cell proliferation or apoptosis, making their role difficult to decipher due to the overwhelming number of conflicting results.

In this review, we discuss the advantages and limitations of current strategies targeting NO/cGMP/PKG signaling in breast cancer, highlighting the therapeutic potential of cGMP hydrolyzing PDE5 inhibitors in a number of preclinical and clinical studies.

## 2. Double-Edged Role of NO/cGMP Signaling in Breast Cancer

Cancer metabolism is governed by intrinsic and extrinsic factors that cooperate to boost cell proliferation. Among other actions, the mechanistic target of rapamycin complex 1 (mTORC1) regulates anabolic pathways and stimulates the synthesis de novo of purine and pyrimidine nucleotides, the RNA and DNA building blocks, by controlling the mitochondrial tetrahydrofolate cycle and by activating glycolysis and the pentose phosphate pathway [25,26,27].

Purine metabolism has aroused great interest, playing an essential role in cancer cell bioenergetics, proliferation and death. The de novo purine biosynthesis and purine salvage pathways serve as nucleotide providers for cell growth (G1), the synthesis of genetic materials (S), division (G2) and mitosis (M), which are made possible through the up-regulation of phosphoribosylpyrophosphate synthetase 2 (PRPS2) activity, an enzyme catalyzing the first reaction in nucleotide synthesis and linked to cancer progression [28]. Cytotoxic drugs targeting nucleotide metabolism are clinically approved. For example, leflunomide, an inhibitor of the de novo pyrimidine synthesis is used in combination with doxorubicin, an antineoplastic drug belonging to the class of anthracyclines, commonly used in hematological and solid tumors (Baxter-Holland & Dass, 2018). Since leflunomide sensitizes triple-negative breast cancer (TNBC) cells to doxorubicin, their association has proved to be more effective for the treatment of TNBC.

Nucleotide biosynthesis is also stimulated by the oncogene c-Myc, again by virtue of the up-regulation of PRPS2 [29].

Consistent with this view, in a recent work, it was reported that several genes encoding for enzymes responsible for nucleotide de novo synthesis, namely Prps2, Ppat, Pfas Gmps, and Gart, were up-regulated in 4TO7^Lung^ cells, murine breast cancer cells with higher lung metastatic behavior, and were positively correlated to cancer metastasis, cell stemness (i.e., CD44 and CD24 positive cells) and worse survival [30]. Thus, beneficial effects were obtained by silencing *prps2* and counteracted by using a cGMP analog, which restored the stemness signature and metastatic potential of 4TO7^Lung^ cells. Intriguingly, the other cyclic nucleotide cAMP did not affect cancer cell stemness or invasiveness. The authors suggested that one of the reasons why these cells may promote cancer cell stemness may be the activation of cGMP/PKG-MEK-ERK signaling pathway.

In the tumor microenvironment (TME), comprising stromal cells, endothelial cells, activated fibroblasts, immune cells and tumor cells themselves, many signaling pathways combine to generate NO in order to promote tumor cell survival and proliferation. High levels of NO in TME can be the result of the up-regulation, following a chronic inflammatory state, of iNOS able to generate a potentially mutagenic/carcinogenic product [31].

Indeed, increased NOS activity is a feature of many tumors, e.g., lung, colon, and head and neck carcinomas [32,33,34,35,36,37] In Chinese hamster ovary (CHO)-K1 cells, treatment with basic fibroblast growth factor (bFGF) induced eNOS-mediated NO generation, which contributed to cell proliferation through the cGMP/PKG axis and ceramide synthesis [38].

A positive correlation between NOS expression and tumor grading was also reported in breast cancer, where grade III tumors showed higher levels of NO compared with grade II tumors [39], as well as in mammary phyllodes tumors, where stromal NOS expression correlated with tumor grade and metastatic potential [40].

Conversely, highly invasive MDA-MB-468 TNBC expressed lower levels of NOS and higher levels of arginase activity modulating polyamine biosynthesis accounting for their rapid proliferation [41]. In premenopausal breast cancer patients (<55 years old), the up-regulation of eNOS in peritumoral microvessels surrounding tumor cells was considered a favorable prognostic factor characterized by low relapse and better survival [42]. Similar conflicting results are likely the result of how cell response to NO/cGMP may vary depending on the stimulus (physiological or pathological), the cell type (stromal cells and tumor cells sensing NO in a different way), NO and cGMP local concentrations, and the experimental conditions used in each study.

Mujoo et al. demonstrated that sGC activators, given alone or in combination with NO donors, were able to suppress tumor growth in ovarian, breast, and prostate cancer cell lines [43]. In fact, in these cells the common downstream effector, cGMP, reduced tumor cell proliferation. Specifically, NO donor NOC-18 and, to a lesser extent, sGC activator BAY41-2272 and cGMP analog 8-Br-cGMP, all elicited the dose-dependent growth inhibition of MDA-MB-468 (breast), SK-Br-3 (breast), OV- CAR-3 (ovarian) and PC-3 (prostate) cancer cells, although this was not always dependent on cGMP activation. However, in another study on human ovarian cancer cells, the cGMP analog 8-Br-cGMP was shown to promote cell viability through the inhibition of caspase3-mediated apoptosis and the degradation of p53, this effect being reverted by cell pre-treatment with the sGC inhibitor ODQ [44]. This tumorigenic activity was also reported in metastatic C3L5 mammary cell line expressing high levels of eNOS, where a NO/sGC/cGMP/ERK signaling cascade was responsible for increased cell motility by virtue of vascular endothelial growth factor (VEGF) and endothelin (ET) activation. This effect was reduced, in a dose-dependent manner, by NOS inhibitor L-NAME or RP-8-Br cGMP, a cGMP antagonist/PKG inhibitor [31].

The ability of NO to induce tumor growth and metastasis was explained by the promotion of angiogenesis and invasive migration, as well as by the up-regulation of cyclo-oxygenase (COX)-2 and heat-shock proteins, and the accumulation of p53 via the suppression of proteasomal degradation. P53 regulates the expression of a number of genes associated with cell-cycle arrest and apoptosis (i.e., p21, BAX, FAS) and its gene expression is one of the factors determining cell sensitivity to NO. Accordingly, at high concentrations, NO is cytotoxic in tumors through p53-dependent or independent apoptosis. The anti-proliferative effect of NOS inhibition was observed in tumor cells, where NOS activity was reported mainly at the mitochondrial level, thus affecting respiratory chain and cell bioenergetics [45]. This finding is in accordance with other studies highlighting the importance of mitochondrial compartmentalization of several enzymes and their substrates in hypoxic tumor environment [46,47].

## 3. Challenges of Targeting cGMP Signaling in Breast Cancer

The extent of cGMP signaling in a specific cell compartment is highly dependent on the expression and selectivity of the PDE isoforms present in that niche, together with the tissue distribution of the ATP-binding cassette (ABCC) transport pumps, such as the multidrug resistance protein (MRP) 4, MRP5, and MRP8, deputed to the cGMP active transport out of the cells [48]. In breast cancer, MRP5 and 8 can also contribute to resistance to tamoxifen and other chemotherapeutics [49]. Interestingly, PDE inhibitors were shown to inhibit several MDR transporters (e.g., ABCC4/MRP4, ABCC5/MRP5, ABCB1/P-gp, ABCG2/BCRP), thus enhancing chemotherapeutic drug accumulation in tumor cells and efficacy [50,51].

Cyclic GMP signaling is differentially activated in all breast tumors, and the amplitude of this signal mostly depends on PDE5 expression. In addition, cGMP levels are controlled by a negative feedback mechanism, wherein cGMP would allosterically bind the GAF domain of PDE5 (at the N-terminal of the hydrolase), stimulate PKG-mediated PDE5 phosphorylation on serine 102 and increase its own degradation [52]. Notably, the PDE5 gene was inversely associated with multiple metastasis suppressor genes (MSGs) (ARHGDIB, BRMS1, CASP8, CD44, CDH2, MAP2K4, MAPK14, PEBP1) in three human breast cancer gene expression cohorts (GSE2034, GSE1456 and GSE26304). MSGs are genes that, once re-expressed, can suppress tumor metastasis through different protective signaling pathways. PDE5 downregulation reduced metastasis in experimental models of breast cancer, although it did not affect cell proliferation [53].

Cyclic GMP-hydrolyzing PDEs represent the common target of many drugs, ranging from nonsteroidal anti-inflammatory drugs (NSAIDs) to PDE5 inhibitors. The latter (i.e., sildenafil, vardenafil, tadalafil,) are mostly known for their clinical use in the treatment of erectile dysfunction [54]. They are also applied in other diseases, such as male lower urinary tract symptoms (LUTS), psoriasis arthritis, nonalcoholic fatty liver disease (NAFLD), liver cirrhosis, pulmonary arterial hypertension (PAH) [55,56,57,58,59,60]. Nevertheless, these compounds, alone or in combination with chemotherapeutics, have been heralded as promising drugs for the treatment of several malignancies, including breast cancer, although their role is yet to be fully clarified [61,62,63,64].

Further molecular pathways are related to the cGMP system. In late 1997, a research group reported the chemopreventive effect of the NSAID, sulindac sulfide, in an experimentally induced tumor model. Notably, sulindac sulfide is administered as a pro-drug, then metabolized to the active metabolite, which inhibits COX-1 and COX-2 enzymes and prostaglandin synthesis, while the other metabolite, sulindac sulfone, is responsible for the inhibition of mammary carcinogenesis induced by low or high doses of l-methyl-l-nitrosurea (MNU) in rats [65]. However, the authors remarked that both sulindac sulfone and sulfoxide were able to inhibit dimetilbenz(a)antracene (DMBA)-induced alveolar nodulogenesis, suggesting that these compounds might exert a chemopreventive effect independently of COX inhibition. Furthermore, they observed that these compounds could preferentially inhibit the growth of cells harboring mutated Ha-ras genes. In a model of rat mammary tumors induced by DMBA, the selective COX-2 inhibitor Celecoxib, given daily in the diet, reduced tumor growth in 90% of the rats [66]. More recently, an overwhelming amount of data have been generated that support the positive correlation between COX-2 up-regulation and premalignant/malignant tumors, the COX2-mediated production of mutagens being the most common hypothesis for its carcinogenic activity. For this reason, it has been pursued as a potential target in the setting of a chemopreventive therapy [67,68,69]. The molecular mechanism underlying the chemopreventive effect of COX-2 inhibitors is the inhibition of COX-2 enzyme activity, the rate-limiting step of the synthesis of prostaglandins and other potentially mutagenic metabolites [70], and angiogenesis [71], both associated with tumorigenesis. These results were corroborated by later studies. In relation to the NO/cGMP system, NO is able to induce COX-2 and prostaglandin synthesis, thus yielding an inflammatory microenvironment prone to change into a tumorigenic environment [31]. Accordingly, in transgenic mice overexpressing COX-2 in mammary glands, the down-regulation of the pro-apoptotic proteins, Bax and Bcl-x(L), and the up-regulation of the anti-apoptotic protein Bcl-2, contributed to reducing programmed cell death and favoring the accumulation of dysplastic/neoplastic cells [72]. Furthermore, the COX inhibitor sulindac sulfide was shown to down-regulate cGMP-selective PDE5 and induce cGMP accumulation and PKG activation, thus leading to apoptosis and cell growth inhibition in human SK-BR-3, ZR75-1, and MDA-MB-231 breast cancer cells (IC50 60–85 μmol/L). This effect, evaluated by means of the early marker of apoptosis, caspase-3/7 activity, was not observed in normal human mammary epithelial cells, wherein cGMP hydrolysis was catalyzed by other PDEs than PDE5. The IC50 value of sulindac sulfide was higher when cells were pre-treated with GC inhibitor, LY83583, or lower following cell treatment with nitric oxide donor, NOR-3, therefore suggesting the involvement of NO/GC/cGMP signaling. This effect was exclusive of cGMP as neither the adenylyl cyclase/cAMP activator (forskolin) nor the cAMP PDE inhibitors (e.g., IBMX, EHNA, zaprinast and dipyridamole) were able to affect tumor cell apoptosis [73].

The same research group, in a later work, specified that the cGMP-mediated inhibition of breast tumor cell growth by sulindac sulfide or known PDE5 pharmacologic inhibitors (e.g., MY5445 and tadalafil) was due to the degradation of nuclear β-catenin following its phosphorylation at the serine 33–37, or threonine 41 residues by PKG and, as a consequence, the suppression of oncogenes regulated by β-catenin, including survivin and vasodilator-stimulated phosphoprotein (VASP). These results were confirmed by PDE5 suppression with siRNA [74]. The nuclear translocation of β-catenin upon Wnt signaling is a well characterized pathway in tumor progression and metastasis [75]. Conversely, phosphorylation at Ser33/Ser37/Thr41, which targets β-catenin for its degradation, and the inhibition of TCF/LEF transcriptional regulation are two events counteracting tumorigenesis. Indeed, the inactivation of β-catenin was also reported in colon cancer cells (HT29, T84, and HCT116), wherein treatment with the PDE5 inhibitor exisulind provoked the enhanced PKG-mediated apoptosis of cancer cells [76].

Notably, MCF-7 cells overexpressing PKGII, after being infected with adenoviral constructs encoding the cDNA of PKGII, and treated with 8-pCPT-cGMP, showed a significant inhibition of epidermal growth factor (EGF)/EGFR-induced MAPK/JNK signaling pathway and, as a consequence, growth arrest [77].

In patients with breast cancer, a positive correlation was reported between the expression of PDE5 and PDE9 and several prognostic factors (tumor grade, stage and lymph node involvement), clinically evaluated by the Spearman test. However, an inverse correlation was found between the same PDEs and patient age; this was likely due to the basal higher expression of these enzymes in younger patients [78]. Accordingly, in lung, prostate, and colon cancers the inhibition of PDE5 by exisulind and sildenafil increased cGMP levels and related cancer cell apoptosis [79].

The protective effect of activated cGMP signaling on human breast cancer cell lines MCF-7 (ER-positive) and MDA-MB-468 (ER-negative) is described in the following two studies. In the first, cell treatment with YC-1 [3-(5′-Hydroxymethyl-2′-furyl)-1-benzyl indazole], an allosteric activator of sGC, caused cell growth inhibition in a dose-dependent manner. The same result was observed after cell treatment with the cGMP analogue and PDE-resistant 8-Br cGMP, corroborating the involvement of cGMP/PKG axis in the apoptotic effect, evaluated by annexin-V/PI staining [78]. In the second study, cell treatment with BAY 73-6691, a selective PDE9 inhibitor, promoted cGMP increase and subsequent apoptosis via caspase 3 activation. The apoptotic effect was more evident in ER-positive cells, MCF-7 [80].

In another study, it was demonstrated that PDE5 was up-regulated in HER2-enriched and triple-negative subtypes, where it carried the most malignant phenotype compared to Luminal B and the Luminal A (ER-positive) subtypes (p = 0.014, HR = 1.2) [81]. In this study, MCF-7 cells overexpressing PDE5 showed higher motility in wound-healing scratch assays and a significant phosphorylation of c-Myc, a well-known oncogene, upon the activation of the Rho family of GTPases.

In summary, targeting the cGMP signaling pathway has demonstrated a great potential in cancer therapy, despite the conflicting findings that can be explained by the complexity of TME, wherein cells release signaling molecules (i.e., NO) that exert opposite effects in relation to extrinsic and intrinsic factors.

Of note, several cancer therapies took advantage of NO- and cGMP-mediated ability to sensitize cancer cells to chemo- and radiotherapy and to induce apoptosis. For example, in colorectal cancers, NO-NSAIDs (hybrid nitrates conjugated to an NSAID) have shown both anti-inflammatory and antiproliferative effects [82]. Despite encouraging preclinical results, NO-NSAIDs drugs revealed severe side effects, from gastro-intestinal ulcers to genotoxicity (see NO-aspirin), which questioned their use in cancer therapy [83]. Another example is the use of PDE5 inhibitors (sildenafil, tadalafil, and vardenafil) in mouse models of colon carcinoma, mammary adenocarcinoma, and fibrosarcoma, where the accumulation of cGMP inhibited myeloid-derived suppressor cells (MDSCs)-elicited immunosuppression, enhanced intratumoral T cell infiltration, and favored tumor regression but not eradication [84].

The use of PDE5 inhibitors has also achieved some success in the context of combination therapy with chemotherapeutic drugs, such as in association with doxorubicin in in vivo models of brain tumors, where they ameliorated drug delivery through the blood brain barrier by selectively increasing tumor capillary permeability and vesicular transport upon cGMP interaction with calcium-dependent potassium (KCa) channels [85], as well as in in vitro breast tumors. Specifically, sildenafil enhanced sensitivity to doxorubicin in p53-mutant MDA-MB231 and p53-null MCF-7/E6 cells and, to a lesser extent, in MCF-7/caspase 3 and 4T1 cell lines, by increasing the apoptotic rate and DNA breaks, i.e., after the phosphorylation of γ-H2AX [86]. Moreover, the chemoadjuvant activity of sildenafil consisted in increasing the Enhanced Permeation Retention (EPR)-based anticancer drug delivery to the tumor tissue, which locally increased drug concentration [51].

The synergistic effect of sildenafil in combination with a chemotherapeutic drug has been evaluated in Poly (ethylene glycol)-poly (lactic acid) (PEG-PLA) micellar formulations loaded with crizotinib, anti-tumoral drugs, and sildenafil. MCF-7 cell incubation with these nanoformulations led to reduced cell viability via increased caspase 3/7 activity after 24 h [87]. Importantly, in this co-treatment using nanomicelles, crizotinib was used at half of the dose used in the free drug assay, suggesting its superior bioavailability and therapeutic efficacy. In line with this finding, Greish et al. reported that female Balb/c mice inoculated with 4T1 cells and treated with a combination of sildenafil and doxorubicin, the latter being loaded in nanoformulations, showed a fivefold reduction of tumor size [88].

Despite this innovative delivery approach, which has already been approved by the FDA and European Medicines Agency, more efforts should be made to study the pharmacokinetic profile and potential off-target effects in vivo before the clinical application of this new formulation.

Furthermore, the therapeutic repurposing of PDE5 inhibitors as chemoadjuvant agents has been corroborated by the synergistic effect of sildenafil and cisplatin on the ROS-mediated apoptosis of human mammary adenocarcinomas cells, MCF-7 and MDA-MB-468 [89]. In both cell lines, co-treatment with cisplatin and sildenafil increased the pro-apoptotic BAX and caspase 3 expression while it reduced the expression of the anti-apoptotic BCL2 only in MCF-7 cells. This synergistic effect was dose- and time-dependent. Similar results were obtained by El-Naa et al. [90] in breast tumors excised from mice receiving a combination of cisplatin and sildenafil. This therapy significantly decreased sub-G_1_- (apoptotic cells) and G_1_-phase cell populations, as well as the expression of VEGF and Ki-67, whereas it increased caspase-3 expression compared with mice receiving cisplatin alone. Indeed, the IC50 of cisplatin alone was 4.43 µg/mL, while the IC50 of the combination of drugs was 3.98 µg/mL. In a recent work, the co-treatment of MDA-MB-231 breast cancer cells with sildenafil and a HSP90 inhibitor, PU-H71 showed a potentiated cytotoxic effect due to decreased HSP90 expression, followed by degradation of protein kinase D2 (PKD2), a protein essential for tumor cell proliferation [91].

Accordingly, in prostate cancer cells, sildenafil increased the doxorubicin-mediated apoptotic effect through the generation of ROS and the activation of caspase-3 and -9 and the down-regulation of the anti-apoptotic Bcl-xL and Bad [92]. In addition, sildenafil may exert a cardioprotective role when administered with doxorubicin, a drug often associated with increased risk of cardiomyopathy and congestive heart failure [93]. Interestingly, the multiple sclerosis drug FTY720 (fingolimod), administered in athymic mice bearing BT474 breast cancer tumors, significantly improved the cytotoxic effect of sildenafil in combination with celecoxib, a COX-2 inhibitor, thus offering proof of the advantages of a multifaceted therapeutic approach to a single disease [94]. In the same study, PDE5 inhibitors (e.g., sildenafil and tadalafil) were shown to synergize with COX-2 inhibitors in blocking tumor growth through activation of CD95 death receptor/JNK and the subsequent induction of autophagy, endoplasmic reticulum stress signaling, suppression of sphingosine-1- phosphate (S1P) signaling and ceramide synthesis, as well as by deregulating several signaling pathways linked to cell proliferation (MAPK/ERK, Akt, mTOR, NFκB). These effects were reverted by cell pre-treatment with NOS-I, L-NAME, thus reflecting the involvement of NO synthesis in the combinatory effect of PDE5- plus COX2-inhibitors. Moreover, PDE5 inhibitors, in association with doxorubicin, mitomycin C, and gemcitabine, were able to induce autophagy through the receptor interacting protein 1 (RIP1) pathway and caspase 8 activated death receptor signaling [95]. The most recent studies about the co-treatment of PDE5 inhibitors and other drugs in several breast cancer models are reported in Table 1.

However, it should be noted that pharmacokinetic profiles vary among different PDE inhibitors [98,99]. When evaluated for plasma pharmacokinetics in healthy subjects, tadalafil has demonstrated a longer half-life (17.5 vs. 4 h) compared to sildenafil and vardenafil, which would allow a single administration daily. Sildenafil has the shortest half-life (3–4 h) among the PDE5 inhibitors and shows a poor bioavailability, of 40%, being metabolized by hepatic CYP3A4/5, CYP3A5, CYP2D6, and CYP2C19. However, there is a risk of accumulation in the use of this drug due to its low clearance [100].

At present, PDE inhibitors are being evaluated in many ongoing clinical trials (Table 2). In a recent work, Huang et al. reported that treatment with PDE5 inhibitors in male patients diagnosed with colorectal cancer reduced mortality (adjusted HR = 0.82, 95% CI 0.67–0.99) and metastasis (adjusted HR = 0.85, 95% CI 0.74–0.98), thus positively affecting patients’ prognosis [101]. Unfortunately, a meta-analysis reported an elevated risk of melanoma and basal cell carcinoma following the chronic use of PDE5 inhibitors, although the cause–effect relationship was not fully proved [102]. They are generally well tolerated, with headaches, flushing, rhinitis, cardiovascular effects, dyspepsia, and vision disturbances being the most common side effects. The off-target effects are likely due to the non-specific binding to PDEs other than PDE5. For example, these drugs might bind PDE6 in the rod and cone cells of the retina, causing altered-color vision, or PDE11 in skeletal muscle, leading to myalgias.

For a thorough discussion of marketed PDE inhibitors in cancer and other diseases, as well as their pharmacokinetic/pharmacodynamic profiles, see [122,123].

## 4. Concluding Remarks

Carcinogenesis is the result of the accumulation of genetic mutations, which allow cells to escape from diverse mechanisms of growth control and programmed death (apoptosis). Extensive research in the oncologic field has been pursued to decipher novel molecular checkpoints able to interfere with signaling pathways dictating cell differentiation, proliferation, and invasion. Notably, the punctual inhibition of these cancer-promoting molecules is a significant challenge regardless of the type of tumor, since the same compound might be tumor-promoting in one cell compartment but also vital in a different niche.

In the intricate network of extracellular and intracellular signaling pathways implicated in carcinogenesis, a critical role is played by cGMP and its upstream (e.g., NO) and downstream (e.g., PKG) effectors, which actively regulate tumor cell microenvironments. Cyclic GMP takes part in a myriad of cell events, including adhesion, proliferation, motility, apoptosis, and energy homeostasis. The amplification of the cGMP signal occurs via three principal mechanisms: the stimulation of cGMP synthesis, the activation of cGMP receptor (sGC), and the inhibition of the cyclic nucleotide hydrolysis by selective PDE inhibitors. The latter are a class of drugs that has been repurposed during the years since their first use in the treatment of angina pectoris to erectile dysfunction, pulmonary arterial hypertension, and cancer.

In this review, we collected the most relevant in vitro and in vivo studies regarding the role of NO/cGMP/PKG signaling pathway in breast tumors. The deregulation of cGMP signaling in breast tumors is likely due to an overall up-regulation of cGMP-hydrolyzing PDE5, a decrease in intracellular cGMP levels due to an overexpression of MRP5 responsible for cGMP efflux, and the down-regulation of PKG, the main cGMP effector. The compartmentalization of cGMP in diverse pockets of the cell serves numerous functions, depending on the identity of the organ/tissue and the patho-physiological stimuli, the cell redox state, the levels of p53 and other transcription factors regulating this nucleotide pool. Normally, this compartmentalization allows the cell to simultaneously respond to extracellular and intracellular signals and is achieved through the presence of MDR pumps that sense excessive cyclic nucleotide levels. Cyclic GMP signaling can be pharmacologically targeted at multiple levels, from NO donors (e.g., SNP) to cGC stimulators (e.g., guanylin, BAY41-2272), PDE inhibitors (e.g., sildenafil, tadalafil), and NSAIDs (e.g., COX-2 inhibitors).

The protective effect of cGMP activation against breast tumor progression was mostly explained by the induction of apoptotic pathways through the activation of caspase-3 and -9, PKG-induced β-catenin phosphorylation and ubiquitination, the down-regulation of oncogenes and anti-apoptotic genes (e.g., cyclin D1, c-myc, survivin), and the inhibition of PDE5. Consequently, PDE5 inhibitors were shown to synergize with anthracyclines (e.g., doxorubicin) and other chemotherapeutics by increasing tumor cell sensitization to drug-elicited apoptosis, decreasing drug efflux through ABC transporters (MDRs), and boosting anti-tumor immune response by virtue of their immunomodulatory activity. The advantage of using PDE5 inhibitors (e.g., exisulind or sulindac sulfone) is that this isoform is responsible for cGMP degradation in neoplastic but not in normal breast cells, thus avoiding a systemic effect. This is of paramount importance in view of the therapeutic manipulation of selected PDE isoforms.

As cGMP signaling alterations occur at early stages of tumorigenesis, targeting cyclic nucleotide signaling may represent an efficient approach in terms of chemoprevention.

The pharmacological manipulation of such a complex and ubiquitous system is challenging in terms of selectivity, given the crosstalk between NO and cyclic nucleotides (cAMP and cGMP) and their multiple ligands, as well as the establishment of compensatory mechanisms when inhibiting one of those pathways.

Furthermore, when translating pre-clinical to clinical data with regard to the use of a combination therapy, the intra- and inter-individual variability in terms of pharmacokinetic and pharmacodynamic parameters (i.e., drug-drug interactions, plasma protein bound, polymorphisms in CYP3A4 and CYP2D6 metabolizing enzymes) must not be neglected, since these may ultimately affect patients’ responses to the drugs.

Overall, the preclinical and clinical data suggest the protective role of PDE inhibitors as adjuvant agents for chemopreventive and chemotherapeutic treatments, due to their ability to facilitate drug transport, inhibit drug efflux (via inhibition of MDR transporters), and enhance apoptosis and anti-tumor immune response through the recruitment of intratumoral T lymphocytes. It is conceivable that the discussed multimodal therapy of PDE inhibitors, NSAIDs, and chemotherapeutics offers greater efficacy while stemming toxic side-effects due to the lower dosages of the single treatments, thereby positively affecting the tolerability profile of the overall therapy. However, the ability of these drugs to attack myriad intracellular targets may lead, in any case, to unfavorable off-target effects that might preclude their use in clinics.

Nevertheless, significant strides remain to be made as the clinical data obtained so far are not sufficient to prove the survival advantage of these compounds and the absence of long-term side effects.

In conclusion, future efforts should be conveyed to thoroughly characterize the different PDE isoenzymes and cGMP pattern in normal vs. tumor cells in order to reconcile the conflicting results and dissect their role as diagnostic and prognostic biomarkers, as well as developing effective cGMP-targeted therapeutics.

## Figures and Tables

**Table 1:** The synergistic cytotoxic activity of PDE5-I in combination with other drugs in in vitro and in vivo models of breast cancer.

Tumor Model	Co-Treatment	Effect	Mechanism of Action	Reference
- Human breast tumor cell lines MDA-MB-231 and ZR75-1	Tadalafil (50 μM) + Sulindac Sulfide (COX-inhibitor) (100 μM)	Tumor growth inhibition	PKG-mediated degradation of nuclear β-catenin and suppression of survivin and vasodilator-stimulated phosphoprotein (VASP).	[74]
- BT549 breast cancer cells	Sildenafil (0.5 μM) + Celecoxib (1 μM) + FTY720 (~50 nM)	Synergistic cytotoxic activity	Suppression of anti-aptotic ERK, AKT, p70 S6K, mTOR, NFκB, activation of JNK, p38 MAPK, ceramide-mediated CD95 activation.	[94]
- f4T1 murine breast cancer cells - female Balb/c mice injected with 4T1 mammary carcinoma cells	- Sildenafil (1–100 µM) + doxorubicin (1 µM) - Sildenafil (1 mg/kg) + doxorubicin (5 mg/kg)	Synergistic cytotoxic activity (reduced tumor volume) and reduction of doxorubicin-mediated cardiotoxic effects	Increased EPR-based anticancer drug delivery	[88]
- SUM149 breast cancer cell line	- MY5445, Sildenafil or Vardenafil (10 µM)	Suppression of cancer stem cells (CSC) subpopulation	Differentiation of CSCs to non-stem-like tumor cells upon cGMP-mediated cAMP/PKA signaling	[96]
- Ehrlich ascites carcinoma cells (EAC) inoculated in female mice - MCF-7 human breast cancer cells	- Sildenafil (5 mg/kg/d) + cisplatin (7.5 mg/kg) on the 12th day after EAC cells inoculation - sildenafil (5, 12.5, 25, and 50 µg/mL) + cisplatin (5, 12.5, 25, and 50 µg/mL)	Synergistic cytotoxic activity	Reduced angiogenesis and proliferation, increased apoptosis: decrease of VEGF, angiogenin and tumor necrosis factor-alpha and Ki-67, increase of caspase-3 expression	[90]
- MCF-7 and MDA-MB-468 human breast cancer cells	Sildenafil (50, 100 μM) plus cisplatin (15 μM and 22 μM)	Synergistic cytotoxic activity	Tumor cell sensitization to cisplatin, increase of ROS accumulation into the extracellular environment, increased apoptosis via activation of caspase 3 and BAX, and decreased BCL2)	[89]
- MCF-7 human breast cancer cell lines	Sildenafil (40.33 μM)/Crizotinib (55.25 μM)- dual-loaded PEG-PLA micelles	Decrease in cell viability	Caspase-3 and caspase-7 activation	[87]
- MDA-MB-231 human breast cancer cells	Sildenafil (10–50 µM) + HSP90 inhibitor, PU-H71 (50 nM)	Synergistic cytotoxic activity	Decreased HSP90 expression, degradation of PKD2 and increased apoptosis	[97]

**Table 2:** Role of PDE5 inhibitors (PDE5i) in several cancer types, and related clinical trials.

Cancer Type	Role of PDE5i	References	PDE Inhibitor	Clinical Trial.Gov
**Breast**	- Apoptosis - ROS generation - Enhancement of cisplatin-induced apoptosis - EPR augmentation - Enhancement of antitumor immune response - Down-regulation of oncogenic Wnt/β-catenin pathway - Improvement in anticancer drug concentration - Chemoprevention	[51,64,74,88,89]	Sildenafil (PDE5i)	NCT01375699
			Pentoxifylline (non-specific PDEi)	NCT03916068 NCT02898376 NCT00022204 NCT00188669 NCT01082003 NCT00583700
**Colon**	- Apoptosis - ROS generation (down-regulation of MCL-1, BCL-XL, thioredoxin and SOD2 - Alteration of normal epithelium (reduction in the proliferative compartment in the colon) - Suppression of inflammation - Chemoprevention	[103,104,105,106]	Udenafil (PDE5i)	NCT00607282
**Hepatoma**	- Enhancement of the therapeutic efficacy of BET inhibitors via Hippo pathway - Restoration of antioxidant enzymes - Enhancement of anti-tumor immune activity	[107,108,109]	Tadalafil (PDE5i)	NCT03785210
			Pentoxifylline (non-specific PDEi)	NCT01149304
**Leukemia**	- Apoptosis - Potentiation of chemotherapeutic-induced apoptosis	[110,111]	Sildenafil, Tadalafil, Vardenafil (PDE5i)	NCT01117142
			Pentoxifylline (non-specific PDEi)	NCT02451774
			Theophylline (PDE3i-PDE4i)	NCT00003808
**Lung**	- Apoptosis - EPR augmentation - Reduction of cancer stemness - Potentiation of chemotherapy - Sensitization of tumor cells to apoptosis	[51,112,113]	Papaverine hydrochloride (PDE10i) Sildenafil (PDE5i)	NCT03824327
			Pentoxifylline (non-specific PDEi)	NCT00752115
			Theophylline (PDE3i-PDE4i) Tadalafil (PDE5i)	NCT01871454
			Caffeine (non-specific PDEi)	NCT01799161 NCT02080078 NCT04069936 NCT01402089
**Lymphoma**	- Inhibition of romidepsin-induced EBV reactivation - Increase of brain vascular permeability to anticancer drugs	[114,115]	Sildenafil, Tadalafil, Vardenafil (PDE5i)	NCT01117142
			Pentoxifylline (non-specific PDEi)	NCT02451774
**Ovarian**	- Enhancement of anti-tumor immunotherapy - Suppression of chemotherapy resistance through reduction of ABC drug efflux pumps ABCB1 and ABCG2	[94,116]	Caffeine (non-specific PDEi)	NCT04718740
			Dipyridamole (PDE3i)	NCT00002487
**Prostate**	- Ameliorates biochemical recurrence-free and overall survival - Regulation in androgen receptor expression and aromatase activity - Enhancement of anticancer drug-mediated tumor growth arrest (in combination with vincristine, docetaxel)	[117,118,119,120,121]	Sildenafil (PDE5i):	NCT00906269 NCT00142506 NCT01996852 NCT01054001
			Tadalafil (PDE5i):	NCT00931528 NCT00215631 NCT00122499
			Udenafil (PDE5i):	NCT03142542
			Papaverine hydrochloride (PDE10i):	NCT00080808

## Supplementary Materials

The following supporting information can be downloaded at: https://www.mdpi.com/article/10.3390/ijms23010262/s1.
