# Supplementary material for: Pros and Cons of Pharmacological Manipulation of cGMP-PDEs in the Prevention and Treatment of Breast Cancer
Source: Int J Mol Sci. 2021 Dec 27;23(1):262. doi: 10.3390/ijms23010262 (PMC8745278; doi:10.3390/ijms23010262)
Supplement: Supplementary file 1 [file ijms-23-00262-s001.zip › ijms-1504796-sup.pdf]

Supplementary Table S1. Tissue distribution, role and relative inhibitors of PDEs.

PDE	Substrate	Tissue expression	Role	Inhibitors	References
**PDE1**	cAMP/cGMP dual specificity. Ca^{2+}/Calmodulin -regulated.	Heart, brain, macrophages, lung, testis, smooth muscle	Vascular smooth muscle contraction and proliferation, neuronal signaling, sperm function.	KS-505a, Vinpocetine, IC86.340, SCH51866	(Beaumont et al., 2014; Dunkerly-Eyring & Kass, 2020; Golshiri et al., 2020; Lefièvre et al., 2002; Orhan et al., 2021; Ozaki et al., 2018; Samidurai et al., 2021; Sharma et al., 2006; Wennogle et al., 2017)
**PDE2**	cAMP/cGMP dual specificity. cGMP-stimulated	Adrenal gland, macrophages, brain, heart, lung, liver, endothelium, platelets	Aldosterone secretion, neuronal signaling, inflammatory-mediated endothelial cell function, regulation of calcium channel in the heart	BAY60-7550, IC933, Oxindole, PDP, EHNA	(Bizzi et al., 2019; Bubb et al., 2014; Chambers et al., 2006; Sadek et al., 2020; Sadhu et al., 1999; Schobesberger et al., 2020; Szarek & Stratakis, 2014; Y.-W. Wang et al., 2021; Weber et al., 2017; Zhang et al., 2017; M.-J. Zhu et al., 2020)
**PDE3**	cAMP/cGMP dual specificity. cGMP-inhibited.	Heart, lung, liver, pancreas, platelets, adipose tissue, inflammatory and immune cells	Vascular smooth muscle contraction, renin release, Insulin signaling, cell proliferation, platelet aggregation, cardiac contractility	Cilostamide, Tolafentrine, Enoximone, Milrinone, Siguazodan, K134, Olprinone, Trequinsin	(Bejaoui et al., 2018; Beute et al., 2021; Coenen et al., 2021; de Havenon et al., 2021; D. Singh et al., 2021; Vinogradova & Lakatta, 2021; Wójcik-Pszczoła et al., 2021)
**PDE4**	cAMP-specific	Heart, Sertoli cells, kidney, brain, liver, lung, inflammatory and immune cells	Vascular smooth muscle contraction and proliferation, immune cell activation, cardiac contractility, neuronal signaling	Rolipram, Roflumilast, Ro20-1724, AWD12281, GSK256066, CBU91, Cilomilast, Denbufylline, Apremilast, Arofylline, Difamilast	(Dong et al., 2021; KleinJan, 2021; Levallet et al., 2008; Ookawara & Nio, 2021; Park et al., 2021; Saeki et al., 2021; Vilhena et al., 2021)
**PDE5**	cGMP-specific	Lung, brain, heart, platelets, penis, vascular smooth muscle, stomach and colon	Vascular smooth muscle contraction, neuronal signaling, platelet aggregation	Sildenafil, Zaprinast, Taldanafil, Exisulind, Vardenafil, Dipyridamole, Mirodenafil, E402, DA8159	(Abdel-Halim et al., 2021; AboYoussef et al., 2021; Cruz-Burgos et al., 2021; Haider et al., 2021; Hoeper et al., 2021; Iwasaki et al., 2021; Kang & Song, 2021; Kopanitsa et al., 2021; Roy et al., 2021; Sakalis et al., 2021; G. Wang et al., 2021; A. Webb et al., 2021; Zhong et al., 2021; G. Zhu et al., 2021)
**PDE6**	cGMP-specific	Photoreceptor	Phototransduction	Dipyridamole, Avanafil, Zaprinast, Udenafil	(Arora et al., 2021; Cote et al., 2021; Leinonen et al., 2021)
**PDE7**	cAMP-specific	Skeletal muscle, heart, kidney, brain, pancreas, T-lymphocytes, liver, testis	Cognitive function, immune cell activation	BRL-50481, ASB16165, IC242, Dipyridamole, GRMS-55	(Y. Chen et al., 2020, 2021; Goto et al., 2010; Hayashida et al., 2021; R. Lee et al., 2002; Świerczek et al., 2021)
**PDE8**	cAMP-selective	Testis, eye, liver, skeletal muscle, heart, kidney, thyroid, ovary, brain, immune cells, colon	Immune cell activation, testis function, production of thyroid hormones	Dipyridamole, PF 04957325	(Epstein et al., 2021; Lounas et al., 2019; Mansuri et al., 2020; Shimizu-Albergine et al., 2012; Tulsian et al., 2021; H. Wang et al., 2008)
**PDE9**	cGMP-specific	Kidney, liver, lung, brain, heart, prostate, spleen	Neuronal signaling	PF04447943, BAY 73–6691, WYQ C28L, SCH51866, CRD-733	(Ceddia et al., 2021; da Silva et al., 2013; Diederen et al., 2007; X.-F. Huang et al., 2018; Rana et al., 2021; Ribaudo et al., 2021; Richards et al., 2021; Wu et al., 2021; Xiao et al., 2021)
**PDE10**	cAMP/cGMP dual specificity	Heart, thyroid, testis, brain	Cognitive functions	Dipyridamole, PQ-10, Papaverine, MP-10, TP-10, ADT 061	(K. J. Lee et al., 2021; Piazza et al., 2020; Titulaer et al., 2019; Y.-F. Yu et al., 2020; Zagorska et al., 2018; Zagórska, 2020)
**PDE11**	cAMP/cGMP dual specificity	Skeletal muscle, prostate, kidney, liver, pituitary gland	Sperm development and function	none	(Pilarzyk et al., 2019; Seftel, 2005; Smith et al., 2021; Wayman et al., 2005)
